# Antibacterial potency of different deposition methods of silver and copper containing diamond-like carbon coated polyethylene

**DOI:** 10.1186/s40824-016-0062-6

**Published:** 2016-07-06

**Authors:** Norbert Harrasser, Sebastian Jüssen, Andreas Obermeir, Ralf Kmeth, Bernd Stritzker, Hans Gollwitzer, Rainer Burgkart

**Affiliations:** Clinic of Orthopedics and Sports Orthopedics, Klinikum rechts der Isar, Technical University of Munich, Ismaninger Str. 22, 81675 Munich, Germany; Experimental Physics IV, Institut für Physik, Augsburg University, Universitätsstr. 1, 86135 Augsburg, Germany; ATOS Clinic, Effnerstr. 38, 81925 Munich, Germany; Clinic of Orthopedics and Sports Orthopedics, Klinikum rechts der Isar, Technical University of Munich, Ismaninger Str. 22, 81675 Munich, Germany

**Keywords:** Implant-associated infections, Diamond-like carbon, Silver, Copper, *Staphylococcus epidermidis*, Antibacterial coating

## Abstract

**Background:**

Antibacterial coatings of medical devices have been introduced as a promising approach to reduce the risk of infection. In this context, diamond-like carbon coated polyethylene (DLC-PE) can be enriched with bactericidal ions and gain antimicrobial potency. So far, influence of different deposition methods and ions on antimicrobial effects of DLC-PE is unclear.

**Methods:**

We quantitatively determined the antimicrobial potency of different PE surfaces treated with direct ion implantation (II) or plasma immersion ion implantation (PIII) and doped with silver (Ag-DLC-PE) or copper (Cu-DLC-PE). Bacterial adhesion and planktonic growth of various strains of *S. epidermidis* were evaluated by quantification of bacterial growth as well as semiquantitatively by determining the grade of biofilm formation by scanning electron microscopy (SEM). Additionally silver release kinetics of PIII-samples were detected.

**Results:**

(1) A significant (*p* < 0.05) antimicrobial effect on PE-surface could be found for Ag- and Cu-DLC-PE compared to untreated PE. (2) The antimicrobial effect of Cu was significantly lower compared to Ag (reduction of bacterial growth by 0.8 (Ag) and 0.3 (Cu) logarithmic (log)-levels). (3) PIII as a deposition method was more effective in providing antibacterial potency to PE-surfaces than II alone (reduction of bacterial growth by 2.2 (surface) and 1.1 (surrounding medium) log-levels of PIII compared to 1.2 (surface) and 0.6 (medium) log-levels of II). (4) Biofilm formation was more decreased on PIII-surfaces compared to II-surfaces. (5) A silver-concentration-dependent release was observed on PIII-samples.

**Conclusion:**

The results obtained in this study suggest that PIII as a deposition method and Ag-DLC-PE as a surface have high bactericidal effects.

## Background

Implant-associated bacterial infections are one of the most serious complications in orthopedic surgery, representing a significant healthcare and economic burden [1]. Management of these infections often requires multiple debridement surgeries, and long-term systemic antibiotic therapy, despite the associated side effects and additional complications [2]. One of the major problems in septic surgery is the formation of biofilm on implanted foreign materials [3, 4]. These extracellular polysaccharide layers impede the activity of the host defenses and antibiotic therapy, leading to a 1000-fold decreased susceptibility to antimicrobial agents, and further promotion of bacterial survival and growth [5]. Once a significant amount of biofilm has formed, eradication of infection is nearly impossible without removing the implant. Therefore, prevention of these infections has an important impact on patient’s morbidity and the cost effectiveness of hospital care [6]. In this context, employment of implant materials or coatings that control infection and biofilm formation are particularly advantageous [7]. This led to the development of antiadhesive and antibacterial surfaces. The first mentioned coatings (e.g. polyethylene glycol, polyethylene oxide brushes) reduce bacterial adhesion by altering the physicochemical properties of the substrate. Thus, formation of protein surface layers (conditioning films) on the implant and bacteria-substrate interactions are hindered [8]. However, the effectiveness of these coatings for reducing bacterial adhesion is very limited and varies markedly depending on bacterial species. On the other hand, non-antibiotic antibacterial coatings actively release bactericidal agents, e.g. silver (Ag)- [9, 10] and/or copper (Cu) [11]. In contrast to antibiotics these ions act more broadly against a wide range of bacteria, and microbes that are not intrinsically resistant [12] will rarely develop resistance [13]. However, there are concerns regarding a possible toxicity of silver-coated medical devices [10]. Cu on the other hand has been shown to possess outstanding antibacterial but nevertheless bio-tolerant features [11, 14]. A problem concerning Ag and Cu as bactericidal agents in coatings is the fact that they can hardly be embedded on wear surfaces, e.g. polyethylene (PE). PE is in widespread use in total joint arthroplasty due to its outstanding mechanical properties as a wear surface and simultaneously its high biocompatibility. On the other hand PE is highly prone to bacterial adherence. In total knee replacement roughly half of the surface is exposed to synovial fluid and in main parts tribologically active. Therefore in septic knee surgery major portions of the susceptible prosthesis are not protected against bacterial reinfection. A potential solution to this problem could be the use of antibacterial-agent-enriched diamond-like carbon (DLC) coatings. DLC coatings can act as local antibacterial agents if release of Ag (Ag^+^)- or Cu (Cu^++^)-ions is provided [15–17], and at the same time exhibit excellent tribological features if used for hip or knee arthroplasty [18–21]. In spite of these promising results, to our best knowledge, comprehensive studies on antibacterial effects of DLC coatings on soft wear surfaces, e.g. polyethylene (PE), comparing Ag and Cu have not been conducted so far. Additionally, data on the use of different deposition methods for DLC coatings and its influence on antimicrobial effects are still lacking.

In this report the antimicrobial effects of Ag- and Cu-incorporated DLC coatings on PE manufactured with different techniques are described. The coatings and films were deposited by two methods of IBAD (plasma immersion ion implantation (PIII) and conventional ion implantation (II)). Bactericidal potency of DLC specimens enriched with Ag or Cu was studied on the surface and the surrounding fluid medium. This study provides valuable information for determining the suitability of DLC-PE enriched with Ag or Cu. Ethics approval for this study was not necessary according to the institutional review board (TU München).

## Methods

### DLC film deposition

**Fig. 1:**
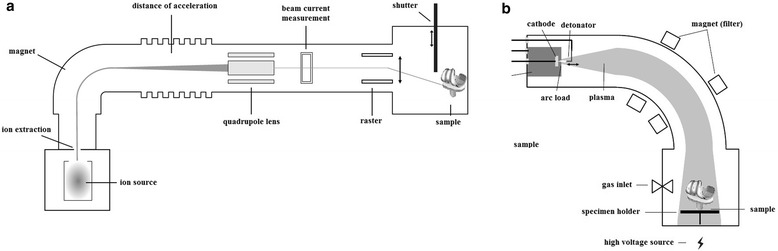
Scheme of deposition methods for ions: **a**) direct ion implantation (II), **b**) plasma immersion ion implantation (PIII); Note: II allows incorporation of ions only on the sample surface struck by the ion beam; PIII allows homogenous incorporation of ions on complex shaped surfaces due to liquid plasma state of the ion beam

To incorporate Ag or Cu homogenously within the DLC matrix of PE-samples modified techniques of ion irradiation of polymers were applied: DLC-processing was achieved by either conventional, direct ion implantation (II) via ion bombardment or plasma immersion ion implantation (PIII) [22]. Both methods are described schematically in Fig. 1. Main disadvantage of conventional II is that only a relatively small part of the surface which is targeted by the beam can be enriched with ions. This makes ion-containing DLC-processing of 3D-surfaces (e.g. joint prostheses) time-consuming and expensive. On the other hand, ion implantation with PIII is easy to perform due to the liquid plasma state of the coating fluid. This allows coating of complex shaped surfaces without major efforts. In contrast to common DLC techniques (e.g., physical vapor deposition) with both methods used in this study the PE-surface is not coated with DLC but rather modified by ion implantation. Due to the kinetic energy of the implanted ions, the polymer surface is modified from crystalline PE to amorphous DLC, while the metal ions agglomerate to nano-particles directly under the surface. In this way, the implantation of ions leads to a wear-resistant, antibacterial PE surface reducing the risk of detachment compared to surface coatings [23].

### Sample features

Study objects were cylindrical substrates (diameter: 10 mm, height: 2 mm; Goodfellow GmbH, Nauheim, Germany) of ultrahigh molecular weight polyethylene (PE). The samples were investigated in different groups with modified parameters of implantation: Firstly, to determine which of the ions (Ag or Cu) exhibits higher bactericidal potency (first group) and secondly, to determine the influence of different deposition methods (second group). All sample features and testing groups are given in Table 1.

**Table 1:** Physical parameters of DLC conversion and antibacterial effect of different surfaces compared to untreated PE

	DLC-processing (implantation energy, fluence)	Surface adhesion [CFU; mean +/− SD]	Bacterial growth of Ag-DLC-PE [log-levels ^a^/ %]^b^	*p*-values	Planktonic growth [CFU/ml; mean +/− SD]	Bacterial growth of Ag-DLC-PE [log-levels^a^ / %]^b^	*p*-values	
Comparison of antibacterial ions deposited with II: Ag vs. Cu	II (Ag): 60 keV, 1x10^17^ cm^−2^	2.6x10^3^+/− 2.5x10^3^	−0.8 / - 85.6 %	<.05*	1.7x10^5^+/− 8.5x10^4^	+0.05 / +13.3 %	>.05*	1st Group
	II (Cu): 55 keV, 1x10^17^ cm^−2^	9.0x10^3^+/− 2.6x10^3^	−0.3 / -50.0 %	<.05*	1,6x10^5^+/− 9.5x10^4^	+0.03 /+6.6 %	>.05*	1st Group
	II (Ag) vs. II (Cu)			<.05			>.05	1st Group
	Untreated PE	1.8x10^4^+/− 9.4x10^3^			1.5x10^5^+/− 2.8x10^4^			1st Group
Comparison of deposition methods: PIII vs. II	PIII (Ag): 5 kV, 1x10^17^ cm^−2^	2,5x10^2^ +/− 1.5x10^2^	−2.2 / -99.1 %	<.05*	1,1x10^4^+/− 2.5x10^3^	−1.1 / - 96.3 %	<.05*	2nd Group
	II (Ag): 10 keV, 1x10^17^ cm^−2^	2,3x10^3^+/− 3.5x10^2^	−1.2 / -92.0 %	<.05*	3,6x10^4^+/− 1.2x10^3^	−0.6 / - 88.0 %	<.05*	2nd Group
	PIII (Ag) vs. II (Ag)			<.05			<.05	2nd Group
	Untreated PE	2.9x10^4^ +/− 2.0x10^4^			3.0x10^5^+/− 6.5x10^4^			2nd Group

^a^log-levels = bacterial counts calculated as shown in following equation: log-levels = log_10_(CFU of Ag-DLC-PE) – log_10_(CFU of untreated PE)

^b^positive values (log-levels/%) express increased bacterial growth on Ag-DLC-PE compared to PE, negative values express reduced bacterial growth on Ag-DLC-PE compared to PE fluence = amount of ions received by a surface per unit area [ions/cm^2^]

* = compared to untreated PE

*PIII (Ag)* plasma immersion ion implantation of Ag-ions

*II (Ag/Cu)* conventional ion-implantation with Ag- or Cu-ions

*CFU* colony forming units

*SD* standard deviation

In the first group Ag-doped (fluence: 1x10^17^ cm^−2^, ion energy: 60 keV) and Cu-doped samples (fluence: 1x10^17^ cm^−2^; ion energy: 55 keV) were assembled for direct comparison of antibacterial activity of these ions. DLC processing was carried out via II of Ag- or Cu-ions. Ion energy was chosen according to previous results, where these enrgies led to a superficial implantation of ions allowing dissolution onto the surface and therefore exhibiting bactericidal effects. With this features the effect of fluence and implantation depth can be minimized so that the intrinsic bactericidal effects of the ions can be estimated. Ion energy of Cu-samples was lower compared to Ag-samples due to the fact that Cu-ions penetrate easier into the PE-surface. Therefore Cu-ions reach the same penetration depth as Ag-ions with lower implantation energies. Based on the findings of the first group the second group was assembled with two different methods of ion deposition: PIII (fluence: 1x10^17^ cm^−2^; pulse voltage: 5 kV) vs. II (fluence: 1x10^17^ cm^−2^; ion energy: 10 keV). Again, different ion energies were applied for either methods to allow equal penetration depth of ions into the samples. Non-modified PE samples served as a control.

After sample preparation incubation for 24 h with *Staphylococcus epidermidis* (ATCC35984) was carried out. Thereafter, antimicrobial effects on the sample’s surface (i.e. bacterial sessile growth) and the surrounding fluid medium (i.e. bacterial planktonic growth) were investigated.

### Evaluation of silver release

Silver release kinetics was evaluated for samples with the highest intrinsic antimicrobial potency, namely Ag-DLC-PE samples deposited with PIII. Sample plates were placed into 10 ml phosphate buffered saline (PBS) and kept sealed for 10 days at 37 °C. Every 24 h PBS was harvested and replaced. For every Ag-enriched sample type (fluences: 1 × 10^17^, 5 × 10^16^ and 1 × 10^16^ cm^−2^) five specimens were investigated. Untreated PE served as control. Analysis of silver release kinetics of the harvested PBS was conducted via ICP-OES (inductively coupled plasma optical emission spectroscopy, Fa. Varian, Vista-MPX, Kleve, Germany).

### Sterilization of samples and sealing of surfaces with paraffin wax

Samples were treated according to a previously described standardized method [24]. Briefly summarized, specimens were rinsed with distilled water for 10 min, air-dried in a laminar flow cabinet and thereafter sterilized with gamma-beam with the dose of 26.5 kGy (Isotron Deutschland GmbH, Allershausen, Germany).

### Bacterial sample preparation

The bacterial strains used in the present study were *S. epidermidis* (ATCC 35984; LGC Standards GmbH, Wesel, Germany) for determination of surface and planktonic growth and a strong biofilm-forming variant of *S. epidermidis* (RP62a; LGC Standards GmbH, Wesel, Germany) for scanning electron microscopy (SEM-) evaluation of biofilm formation on the samples. These strains are of major clinical importance in implant-associated infections [25, 26]. Test strains were routinely cultured in Columbia Agar with 5 % sheep blood (*S. epidermidis,* ATCC 35984) or Trypticase™ Soy Agar (*S. epidermidis,* RP62a) (Becton Dickinson, Heidelberg, Germany) at 37 °C overnight before testing. Bacteria were then harvested by centrifugation, rinsed, suspended, diluted in sterile phosphate buffered saline (PBS) and adjusted by densitometry to a MacFarland 0.5 standard (MacFarland Densimat™, BioMérieux, Marcy l’Etoile, France). To control bacterial concentration, 100 μl of each suspension was again cultured for 24 h at 37 °C. After 24 h serial dilutions of this suspension were plated on Colombia-Agar. The colonies were counted and colony numbers calculated accordingly. For the study every suspension with its known bacterial concentration was diluted with DMEM + 10 % FCS to reach the targeted value for bacterial concentration (10^5^ CFU/ml). Sample plates with paraffin-coated lower surfaces were placed in 24-well culture plates and 1 ml of 10^5^ CFU/ml bacterial suspensions were added. Incubation of the well plates was conducted for 24 h at 37 °C.

### Microbiological analysis

Bacterial surface adhesion was evaluated by determining bacterial concentration on the specimen. Bacterial planktonic growth was measured in the growth medium. For every group four independent testing runs with four different samples were conducted. Therefore, altogether 16 samples were tested for every group.

### Determination of bacterial growth on the sample surface

**Fig. 2:**
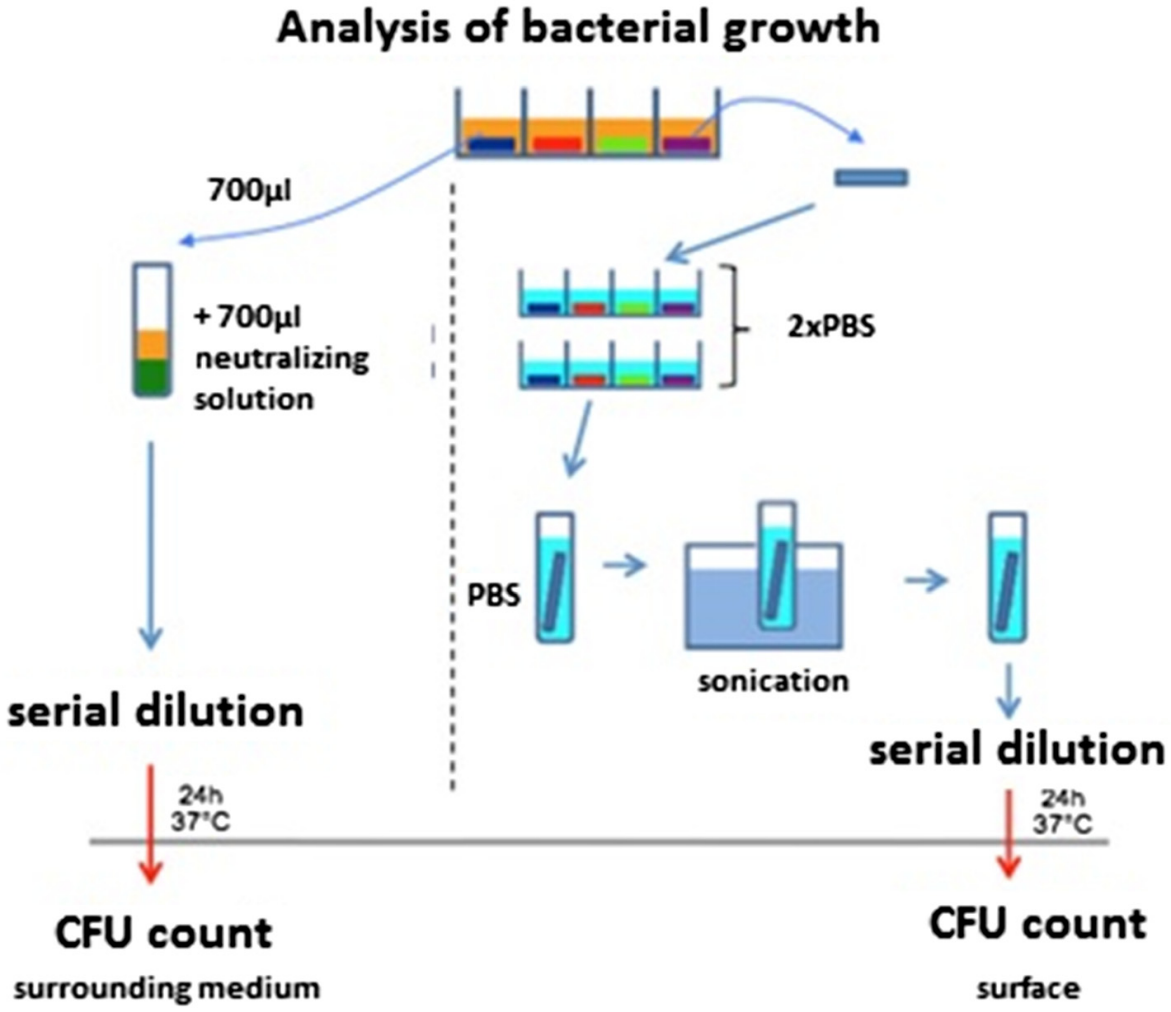
Analysis of bacterial growth (CFU: colony-forming units)

Colonized sample plates were removed from the wells with a sterile forceps, carefully rinsed twice with sterile PBS, transferred to vials containing 3 ml of sterile PBS and sonicated for 7 min (Elmasonic S60H, Elma, Singen, Germany) to remove adhering bacteria. 100 μl of the fluid were aspirated, plated on Colombia Agar at 37 °C for 24 h and quantified after incubation (CFU/ml, Fig. 2).

SEM-analysis was conducted semiquantitatively to evaluate inhibition of biofilm formation. SEM-images were compiled of native DLC coated PE samples and Ag-DLC-PE samples treated with II and PIII. Biofilm formation was quantified in five categories: (1) no biofilm formation, (2) biofilm covering less than 25 % of the surface, (3) biofilm covering between 25 and 75 % of the surface, (4) biofilm covering more than 75 % of the surface, (5) biofilm formation covering the entire surface. Two different observers (NH, SJ) graded five randomly chosen fields of every sample in three runs.

### Determination of bacterial planktonic growth

A 700-μl volume of each well was supplemented with 700 μl neutralizing solution as described by Tilton: 1,0 g sodium thioglycolate + 1,46 g sodium thiosulfate in 1.000 ml deionized water [27]. The neutralizing solution acts as an inhibitor for reminiscent metal toxicity on bacteria. The suspension was plated on Columbia Agar after serial dilutions and incubated at 37 °C for 24 h. Thereafter, CFU were quantified and extrapolated to CFU/ml (Fig. 2).

### Statistics

All results are presented as means ± standard deviation. Statistical significance was computed using non-parametric methods and the method of closed testing procedure (Kruskal-Wallis and Mann–Whitney *U* test). *P* < 0.05 was considered statistically significant. Statistical tests were performed with use of SPSS (version 20.0; Chicago, Illinois). Statistical analysis was conducted per consultation with the Institute of Medical Statistics and Epidemiology (Klinikum rechts der Isar, Technische Universität München, Munich, Germany).

## Results

### Antimicrobial effect of Cu- and Ag-DLC-PE with equal penetration depth of ions in the surface layers (ion energy: 55 keV for Cu and 60 keV for Ag) and equal fluences (1x10^17^ cm^−2^)

**Fig. 3:**
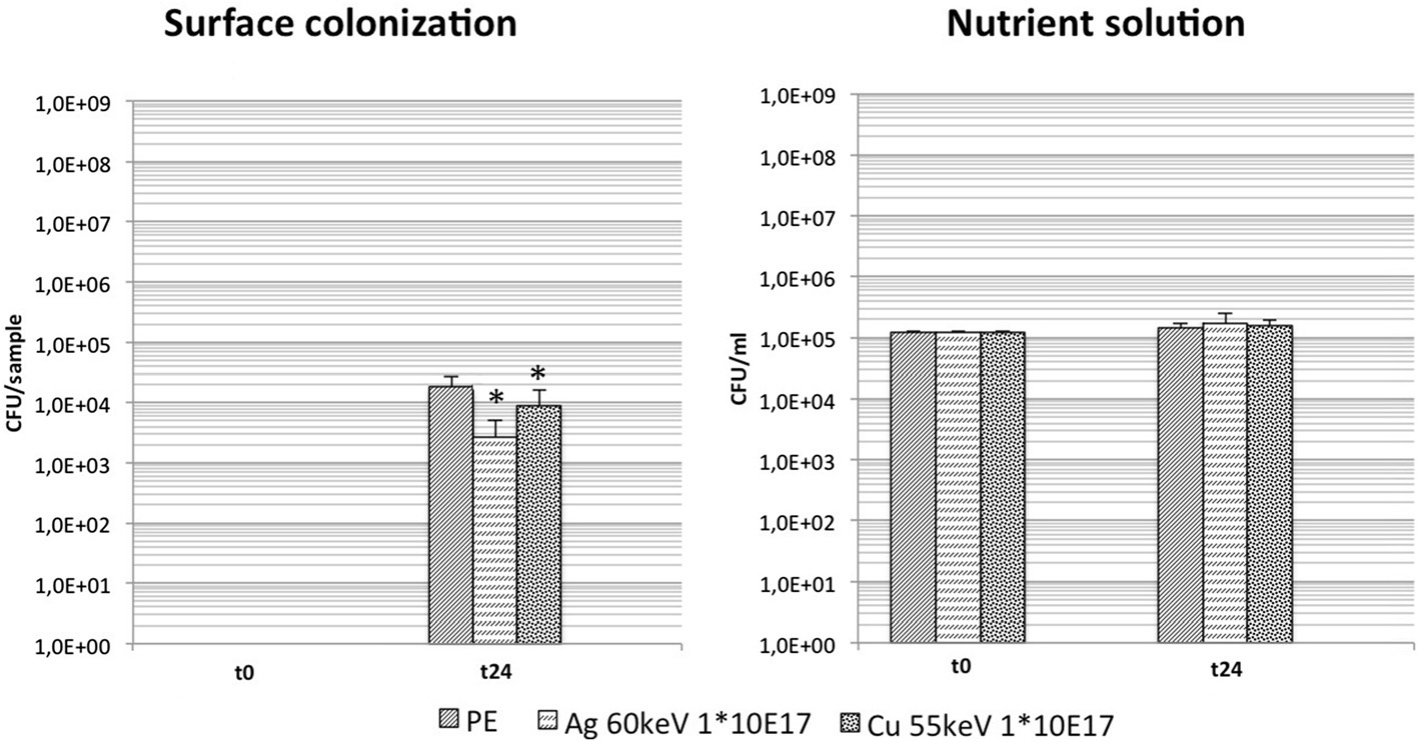
Bacterial growth of *S. epidermidis* in the Cu- and Ag-DLC-PE testing group 1 with comparison of bactericidal potency of Ag- and Cu-ions (t = 0: before incubation; t = 24 h: after incubation); * = *p* < .05 (compared to untreated PE)

Compared to untreated PE on Cu- and Ag-DLC-PE samples a significantly decreased bacterial growth was evident (Table 1, Fig. 3). Comparison of Cu- and Ag-DLC-PE samples among each other revealed a significant reduction of bacterial surface growth on Ag-DLC-PE samples. Analysis of planktonic growth in the supernatant growth medium showed no significant antibacterial effects neither for Cu- nor Ag-DLC-PE samples (Table 1, Fig. 3). Due to superior bactericidal effects of Ag-DLC-PE compared to Cu-DLC-PE further testing was only conducted with Ag-specimens.

### Antimicrobial effect of Ag-DLC–PE processed with different deposition techniques (PIII vs. II) and equal fluences (1x10^17^ cm^−2^)

**Fig. 4:**
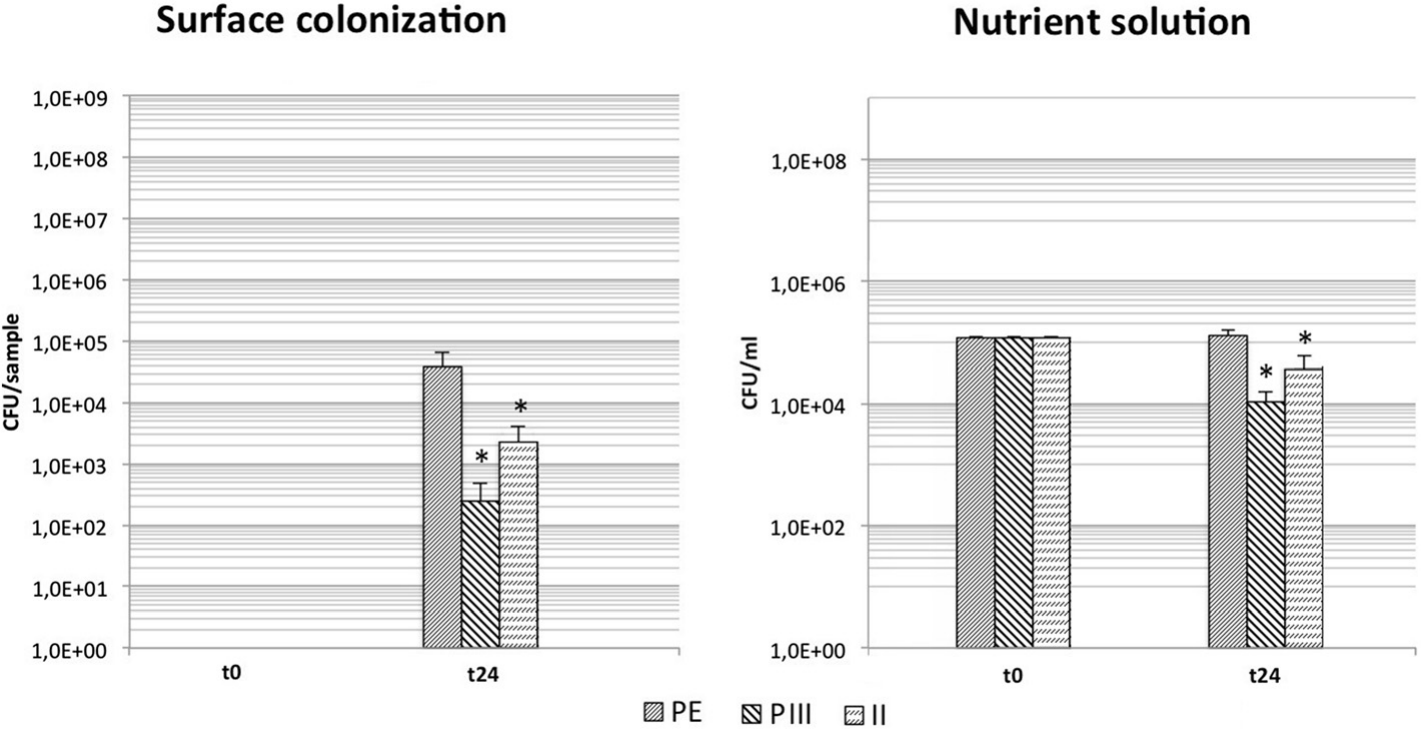
Bacterial growth of *S. epidermidis* in the Ag-DLC-PE testing group 2 with comparison of different deposition methods (t = 0: before incubation; t = 24 h: after incubation; PIII: plasma immersion ion implantation; II: direct ion implantation); * = *p* < .05 (compared to untreated PE)

Samples treated with PIII and II showed both a significantly decreased bacterial surface adhesion compared to PE by 2.2 and 1.2 log-levels respectively. Comparison of PIII and II revealed a significant reduced amount of bacteria for PIII-samples. Analysis of planktonic growth showed again significantly reduced bacterial concentrations for either deposition techniques. Similar differences were found for bacterial concentrations in the surrounding medium (Table 1, Fig. 4).

### Surface biofilm formation in scanning electron micrographs

**Fig. 5:**
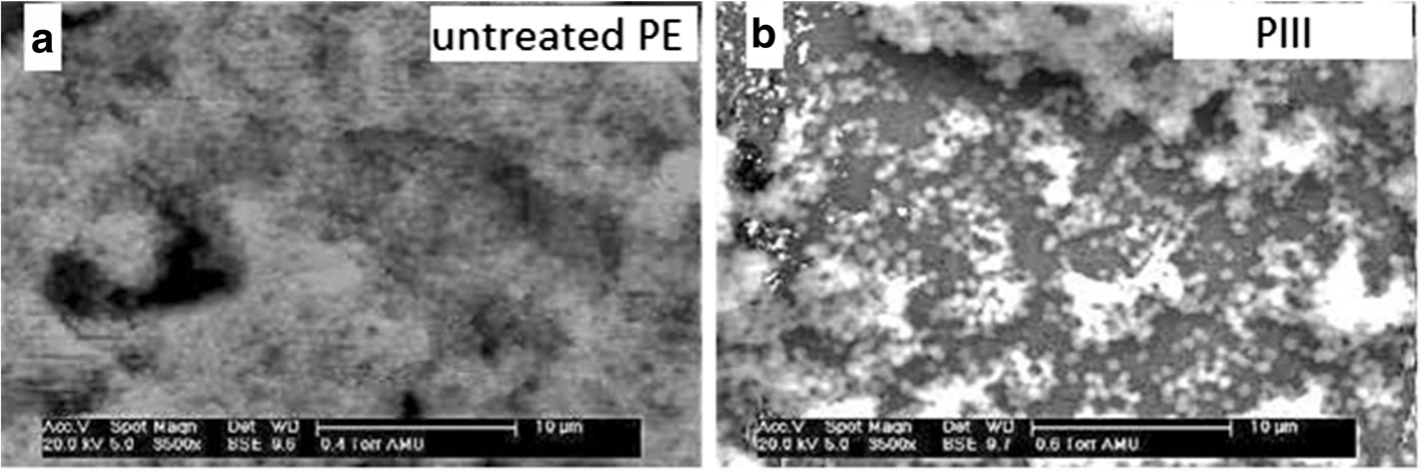
SEM-images to exemplify biofilm formation on different polyethylene surfaces. Homogenous biofilm grade 5 after incubation with *S. epidermidis* on native PE (**a**), reduced biofilm grade 3 on Ag-DLC-PE processed with PIII (5 kV, 1x10^17^ cm^−2^, **b**)

Biofilm formation was ubiquitous and graded type 5 on all native PE samples. The entire specimen surfaces were covered with thick layers of *S. epidermidis*. Ag-DLC-PE samples treated with II on the other hand showed biofilm inhibiting effects with at the most rare spot-like biofilm formation. Average grading for this group were type 4. Samples treated with PIII showed less biofilm formation compared to II-samples. Average grading for this group were type 3 (Fig. 5).

### Silver release kinetics

**Fig. 6:**
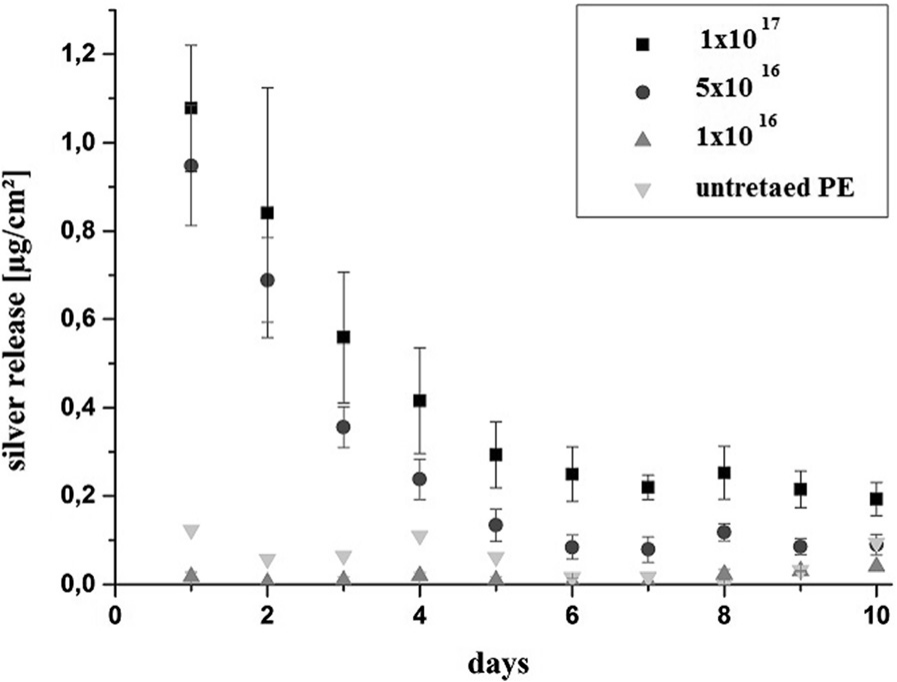
Silver release kinetics of different Ag-DLC-PE (deposition method: PIII; fluence: 1 × 10^17^ cm^−2^, 5 × 10^16^ cm^−2^, and 1 × 10^16^ cm^−2^) and untreated PE samples

Silver release kinetics was evaluated for samples which provided the highest bactericidal potency (PIII-samples). Ag concentration of untreated PE throughout the 10 days was considered as the lower detection limit. Due to high sensitivity of the method values were not zero for these specimens. Ag release of specimens with fluences of 1 × 10^17^ cm^−2^ and 5 × 10^16^ cm^−2^ showed an exponentially decrease up to five days from the beginning of the test (Fig. 6). Thereafter a steady state with minimal decrease of Ag release was achieved. Ag release of Ag-DLC-PE with a fluence of 1 × 10^16^ cm^−2^ was equal to the values of untreated PE and therefore below the lower detection limit.

## Discussion

Since the first applications of surgically-implanted materials in humans, bacterial infections have represented a common and challenging problem [1, 25]. More than 2.6 million orthopedic implants are performed annually in the United States, hence the incidence of implant-associated infections is also increasing [28]. Most important in the pathogenesis is the colonization of the device surface by formation of a biofilm [9, 29–31], at which Staphylococci and Streptococci are most frequently implicated as the etiologic agents [25, 32]. Recent strategies to lower peri-implant infection rates are based on the primary prevention of bacterial adhesion by non-adhesive coatings [33, 34] or impairment of bacterial survival and biofilm formation by surface coatings releasing non-antibiotic organic [35–37] and inorganic agents like Ag^+^, Cu^++^ or nitric-oxide [12, 38–40]. To our best knowledge, few attempts have been conducted so far to apply Cu- or Ag-DLC coatings on PE surfaces [24]. DLC surface modifications could be promising, based on the finding that DLC applied at PE is known to exhibit excellent wear behavior [18–20]. A previous study described significant antibacterial potency of Ag-DLC-PE [24], whether DLC coatings enriched with Cu provide the same ability is still unclear. Additionally, no data are available regarding comparison of different DLC deposition methods.

Ag seems to be of outstanding value in the prevention and treatment of implant associated infections [41–43]. Ag acts by binding to membranes, enzymes and nucleic acids. Consequently the respiratory chain is inhibited and therefore the aerobe metabolism of microorganisms disturbed [9]. Bacteria are quite susceptible to Ag with only negligible possibility of intrinsic resistance [12]. On the other hand, possible toxicity of silver-coated devices is still on debate, which limits its clinical use [10]. Therefore, in the present study one sample series was conducted with Cu-doped DLC coatings since some authors found Cu-ions having an outstanding position as an antibacterial but nevertheless bio-tolerant additive to coatings [14]. Besides these advantages a major disadvantage is the fact that Cu is difficult to implant on hard surfaces, e.g. titanium. The reason is its low solubility in ethanol-based solutions so that assembly of high dosage colloidal solutions for dip-coating is not possible. Cu highly tends to agglomerate in polyvinylpyrrolidone-matrix [44]. This would be limiting in the manufacturing process of DLC coated joint prostheses.

Our results demonstrated minor antibacterial effects on the surface of Cu- compared to Ag-DLC-PE samples (Table 1, Fig. 3). This finding was similarly described on other Cu-containing materials by other investigators [39]. We implanted Cu-ions in the same depth of the samples as Ag-ions. This is crucial in the assessment of antibacterial effects. If ion deposition within soft surfaces is performed with high energies (>80 keV) a rather deep deposition and concomitant slow or missing dissolution of ions onto the surface and into the surrounding medium is achieved. Consequently low bactericidal activity of these samples has to be expected [24]. Due to inferior antibacterial effects of Cu compared to Ag further testing was only conducted with Ag-DLC-PE.

Another finding in the present study was a deposition-depending antibacterial effect of Ag-DLC-PE. A wide variety of techniques have been employed for the synthesis of DLC coatings [19]. Among them, ion beam assisted deposition (IBAD) has great advantages for biomedical applications. It can produce thin films at low substrate temperature suitable for the majority of biomedical materials [45]. The most common used techniques for DLC processing are plasma-based, e.g. chemical vapor deposition (CVD) or physical vapor deposition (PVD) [46]. These methods are merely deposition techniques resulting in adhesive problems due to high internal stresses of DLC layers [47]. The methods of DLC processing in the present study (PIII and II) provide penetration of ions into the PE and concomitant DLC modification of these superficial layers. This fact diminished adhesion problems [48]. In this context, PIII is faster and more cost-effective compared to conventional II and allows DLC coating of complex-shaped surfaces, e.g. joint prostheses [49]. The resulting film properties after PIII treatment should be comparable to those achieved by direct II. On the other hand a clear superiority regarding the bactericidal potency of PIII-samples compared to II-samples was found in the present study. A possible explanation is a quicker dissolution of Ag-ions from the surface of PIII-samples. Therefore, release kinetics of Ag was investigated for these specimens. A high and earlier peak of initial dissolution of Ag for samples with high fluences (≥5 × 10^16^ cm^−2^) was found (Fig. 6). This “hit-hard-and-early-“effect is certainly crucial for the strong bactericidal potency of these coatings. In this context, a large clinical trial revealed no significant differences between silver-coated and uncoated medical devices [50]. One reason for this finding is that the tested coatings did not actively release silver ions. On the other hand, materials that actively release silver in the surrounding medium however have exhibited strong antibacterial activity [12]. Regarding the results of the present study, it is conceivable that samples with equal fluences but deposited with II would have had a lower peak of Ag release. This results in lower bactericidal potency within the first days due to lack of high concentrations of antibacterial ions on the sample’s surface and the surrounding medium. The fact that relatively low concentrated (fluence < 5 × 10^16^ cm^−2^) Ag-samples deposited with PIII did not release Ag can be explained with the “catching-effect” of low amounts of Ag in the polymer matrix [1]. In these circumstances no Ag-nanoparticles are formed and therefore Ag is trapped in superficial PE-layers without the possibility of dissolution.

This study involves several limitations. First, only two bacterial strains were used. Although the investigated strains are of major importance in periprosthetic joint infections, antibacterial effect against other bacteria has to be investigated in future studies. In fact, several studies confirmed even higher bactericidal potency of Ag against Gram-negative compared to Gram-positive bacteria [51, 52]. Second, Cu was only used in the first group. It remains unclear, whether Cu deposited with PIII would lead to increased antibacterial potency in these samples. However, antibacterial effects are caused by the intrinsic activity of the ion and this has been shown to be higher for Ag- compared to Cu-ions. Third, antibacterial effects on the sample surface could be supported by antiadhesive features of DLC alone. A significant antibacterial effect of DLC-PE without integrated Ag/Cu, on the other hand, could be ruled out in our previous experiments [53]. Fourth, no influence of Ag-DLC on osseointegration was investigated. A negative effect on eukaryotic cells in this context could be of major interest in the clinical use of this antibacterial coating even though PE is not used with direct bone contact. However, further investigations are needed in order to clear whether the antibacterial effect of Ag-DLC-PE surfaces is sufficient to avoid implant infection in-vivo.

## Conclusion

Taken together, our findings strongly support further investigation of Ag-DLC conversion of PE manufactured with PIII for prophylaxis of implant-associated infections. Antibacterial effectiveness of Ag-DLC-PE has been demonstrated. The suitability of this surface modification for biomedical applications will be confirmed by future studies.

## Abbreviations

Ag, Silver; Ag+, silver ion; Ag-DLC, silver incorporated diamond-like carbon coating; Ag-DLC-PE, silver incorporated diamond-like carbon coating on polyethylene; Cu, copper; Cu-DLC-PE, copper incorporated diamond-like carbon coating on polyethylene; DLC, diamond-like carbon; DLC-PE, diamond-like carbon coating on polyethylene; PE, polyethylene; PJI, periprosthetic joint infections
